# Extended Salvage Pelvic Lymph Node Dissection in Patients with Recurrent Prostate Cancer

**DOI:** 10.1155/2014/321619

**Published:** 2014-02-12

**Authors:** Daniar K. Osmonov, Alexey V. Aksenov, Annkathrin Boller, Almut Kalz, Diana Heimann, Isa Janssen, Klaus-Peter Jünemann

**Affiliations:** Department of Urology and Pediatric Urology, University Hospital Schleswig-Holstein, Campus Kiel, Arnold-Heller-Stra**β**e 7, 24105 Kiel, Germany

## Abstract

*Background*. Treatment of patients with a biochemical recurrence (BCR) of prostate cancer (PCa) is generally difficult and without valid treatment options. Since 2004 we have been developing therapeutic possibilities for these patients. *Methods*. We retrospectively analyzed a cohort of 41 patients with a BCR of PCa and a mean followup of 40.3 ± 20.8 months. Group 1 (*n* = 10): salvage radical prostatectomy (sRP) with SePLND (salvage extended pelvic lymph nodes dissection) (initial treatment: combined brachytherapy). Group 2 (*n* = 22): SePLND (initial treatment: radical prostatectomy (RP)). Group 3 (*n* = 9): SePLND (initial treatment: RP and adjuvant radiation therapy (RT)). We observed PSA, PSA-velocity, localization of LNs and LNs+, BCR-free period, and BR (biochemical response). *Results*. Group 1: 60% with BCR-freedom (mean 27.2 months). Group 2: 63.6% with BCR-freedom (mean 17.5 months). Group 3: 33.3% with BCR-freedom (mean 17.6 months). In total, BCR-freedom was observed in 23 of 41 patients (56.1%) after salvage surgery. 75.6% of all patients showed a BR. 765 LNs were removed and 14.8% of these were LN+. *Conclusions*. The BCR-free period and BR are comparable in all three groups. Sensibility to ADT can be reestablished and prolonged as a result of SePLND. Multicenter studies are needed for a reliable output.

## 1. Introduction

Over the last years, a number of studies have highlighted the significance of primary pelvic lymph node dissection (PLND) in patients undergoing radical prostatectomy (RP) [1, 2]. Improved knowledge of the pathways of lymphatic spread of metastases and detailed clinical observations have made us pay more attention to primary PLND.

According to our recent results, we believe that extended pelvic lymph node dissection (ePLND) should be performed in all patients with intermediate and high risk (according to the D'Amico classification of PCa) [1]. Moreover, the standard technique of ePLND needs to be revised and supplemented by additional dissection areas such as the triangle of Marcille, the sacral lymph nodes (LNs), and the preprostatic area [1, 3].

In general, the number of patients undergoing salvage treatment of PCa is very small and valid analysis is possible only in multicenter studies. However, despite being a single center, we have been able to involve a cohort of 41 patients over the last 6 years, which has encouraged us to standardize the surgical technique and to expand the indication for salvage surgery.

Does this invasive type of surgery lead to equally successful clinical outcomes in patients with recurrent cancer? There are only a few studies in the literature concerning the clinical outcome of salvage radical prostatectomy (sRP) and salvage extended pelvic lymph node dissection (SePLND) in patients with recurrent PCa compared to a rather large number of studies on salvage radiation therapy (sRT) [4, 5].

There are also no reliable definitions of PCa progression and thus for PCa relapse treatment. It seems to be an individual decision by the urologist if and when androgen deprivation therapy (ADT) is initiated.

The latest and largest study of the Munich Cancer Registry has demonstrated the relevance of combined RP and PLND. Among 13805 patients 938 (10.2%) had LN+ status. 688 LN-positive patients underwent RP and 250 LN-positive patients did not. Patients who underwent both RP and PLND showed a relative survival (RS) rate of 86.2% after 10-year follow-up, while patients who only underwent PLND had a RS of only 40.5%. In other words, patients who also underwent RP had a twice as high chance of RS compared to those that did not [6].

Salvage RP was performed and evaluated in patients with recurrent PCa after radiotherapy. Five years after sRP, 48% of the patients were free from biochemical recurrence (BCR) and, 10 years after the surgery, 37% were still BCR-free [7].

There are only few studies that analyze oncological outcomes of patients who underwent SePLND with or without sRP. These multicenter studies include only a limited number of patients. Limitations for advanced studies consist in the still unclear indications for salvage treatment and a general anxiety regarding possible complications [8, 9].

One of the largest prospective analyses of salvage PLND impact on the prognosis of patients with BCR and nodal pathologic [10] choline PET/CT scan after RP includes only 72 patients. 56.9% of patients achieved BCR. The 5-year BCR-free survival rate was 19%. Preoperative PSA was <4 ng/mL, time to BCR <24 months, and negative lymph nodes at previous RP represented independent predictors of BCR. The 5-year cancer-specific survival was 75% [11].

The study presented is a single-center retrospective analysis of surgical salvage procedures (SePLND and sRP) in patients with PCa recurrence. 41 patients from our department were included in this study. The inclusion criteria were histologically proven PCa, BCR, and/or a suspiciously low PSA-doubling time (DT), with no evidence of bone metastases at the time of salvage treatment. A facultative criterion was a PET-CT morphological evidence of lymphadenopathy. These patients underwent salvage therapy of PCa (sRP, SePLND, and sRT).

## 2. Materials and Methods

**Figure 1:**
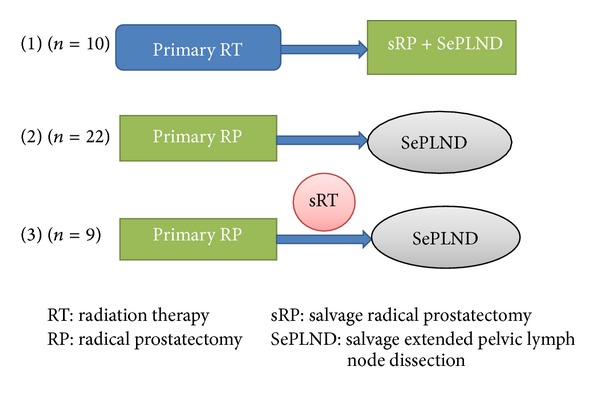
Schematic sequence of treatment options by groups (*n* = 41).

A cohort of 41 patients who underwent salvage treatment between January 2004 and June 2011 at our department has been included in this study.  Figure 1 shows a schematic sequence of the treatment procedures in each group.

 Group 1: combined sRP + SePLND after primary radiotherapy (RT) (*n* = 10).
 Group 2: SePLND after primary RP (*n* = 22).
 Group 3: SePLND after sRT and primary RP (*n* = 9).

There is still no definition of PCa relapse or progression. Criteria of salvage treatment valuation are also not clear. We defined PCa progression as a PSA cutoff of ≥0.5 ng/mL and/or PSA-DT <6 months; in other words, we defined progress of the disease as a biochemical recurrence. A facultative criterion was a PET-CT morphological evidence of lymphadenopathy. We did not perform any needle biopsy before proceeding with salvage surgery. All patients underwent salvage extended PLND (SePLND); in 10 radiation-recurrent cancer patients the procedure was combined with sRP.

11 (36.7%) of the primary RP operations in Groups 2 + 3 (*n* = 30) were performed at our institution according to the classic technique [10] and were supported by PLND, mostly ePLND [12]. The other 19 (63.3%) patients underwent RP/PLND ex domo; the exact number of removed LNs during primary RP/PLND is mostly unknown, 3 of these did not undergo PLND, and all the others underwent limited PLND. These patients came to us for a second opinion after BCR occurrence.

We examined selected patients with BCR and positive PET-CT scans but without bone metastases. SePLND was performed according to our recommendation and after a signed patient informed consent. All patients were duly informed about this type of extended salvage surgery and the advisability of removing a maximum amount of LNs during surgery due to the prostate lymph drainage features and the risk of the existence of micrometastases even in PET/CT-negative locations. The patients were also informed about possible perioperative complications and the possibility of overtreatment, as well as about the lack of survival data and the uncertain success rate of SePLND. None of the patients refused SePLND, which is performed as a standard procedure at our institution.

We evaluated the efficiency of treatment on the basis of the following indicators: stage of disease by TNM classification, Gleason score (GS), type and dose of RT, initial PSA (iPSA), PSA-DT both before salvage therapy and after, the presence/absence of ADT, the dynamics of the PSA for the entire observation period, duration of the BCRF period (months), BR (biochemical response) using Kaplan-Meier curve, the presence/absence of distant metastasis, and the interval between the stages of treatment (primary and salvage) as well as localization and number of metastases in the removed LNs. D'Amico classification was used to define the different risk categories.

**Figure 2:**
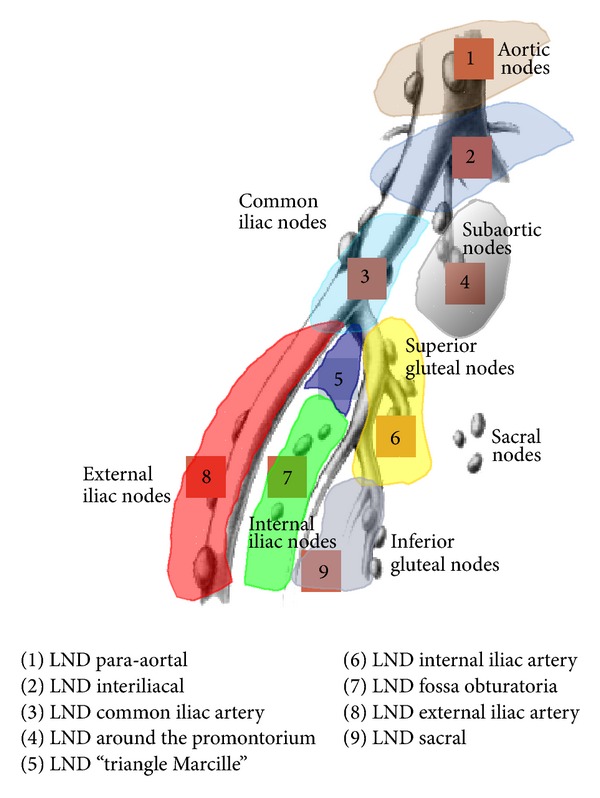
Location of LNs removed during SePLND.

If BCR was diagnosed, we performed a choline PET-CT and bone scan. Patients with bone metastasis were excluded and treated by ADT. ePLND was performed in all patients, regardless of whether PLND was included in the RP or whether it was an independent salvage intervention. There was a standard transperitoneal approach, schematically presented in Figure 2. We performed SePLND in the same way that has recently been described for primary ePLND, including additional dissection areas, which is the standard procedure at our institution [1].

The LN dissection areas were as follows: (1) para-aortal, (2) interiliacal, in the area between the right and left common iliac artery, (3) in the region of the common iliac artery on both sides, (4) around the promontorium, (5) in the presciatic area or the “triangle Marcille,” (6) in the region of the internal iliac artery, (7) in the fossa obturatoria, (8) in the region of the external iliac artery, and (9) in the sacral area (Figure 2).

We also tried to standardize SePLND in view of the operative limitations of salvage surgery due to the scar formation after primary therapy and the presence of easily damageable structures like pelvic vessels and nerves.

We performed SePLND as follows. Choosing a transperitoneal access, we defined landmarks such as the iliac vessels before beginning with the dissection. The ureter was identified and separated carefully from the surrounding tissue. LN dissection was then performed systematically from top downwards. Small or medium clips were used to avoid extensive ligation. We used the harmonic scalpel to seal the LN vessels and to shorten the operation time.

## 3. Results

The age of the patients at the time of primary treatment was 62.3 ± 8.7 (49–74); at the time of salvage treatment it was 66.1 ± 6.5 (54–78).

According to the D'Amico risk classification, there were 3 (7.5%) patients with low-risk cancer, 13 (32.5%) patients with intermediate risk, and 24 patients (60.0%) with high-risk cancer. Patients with low- and intermediate-risk cancer predominated in Group 3, while in Groups 1 and 2 there was a majority of high-risk patients.

Stage *T*_2_ was diagnosed in 17 (42.5%) patients, with a distribution of 3-5-9 (*T*_2*a*_-*T*_2*b*_-*T*_2*c*_) patients, respectively. Stage *T*_3_ was diagnosed in 21 (52.5%) patients with an equal distribution of 10 and 11 (*T*_3*a*_-*T*_3*b*_) patients, respectively.

The three patient groups also differed regarding the average iPSA-levels: in Group 1 the average iPSA was 33.2 ng/mL, in Group 2 it was 19.0 ng/mL, and in Group 3 it was 12.1 ng/mL.

The distribution of patients regarding their primary Gleason score (GS) showed that the predominant GS was 7 (43.9% of patients), then GS 9 (22.0%), GS 8 (17.1%), GS 6 (9.8%), and GS 10 (2.4%). In two patients (4.9%), the GS could not be determined according to the histological report. Only by relying on this indicator it can be said that patients with low risk amounted to no more than 9.8%, otherwise, the patients with intermediate and high risk were dominated.

**Figure 3:**
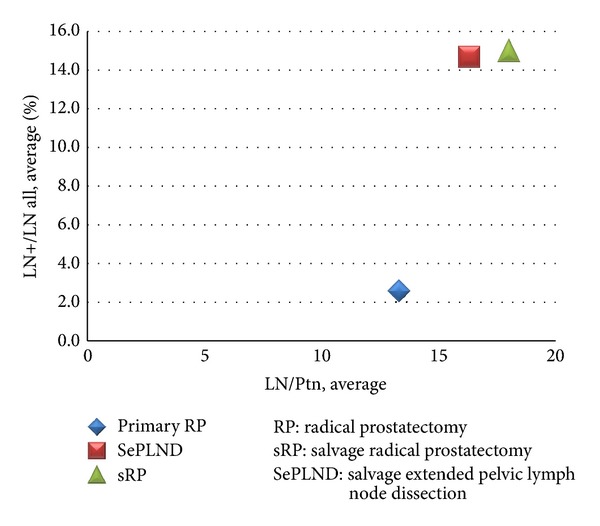
Correlation between number of removed LNs and number of LN+.

In total, of the 765 LNs which were removed during salvage surgery, 113 were diagnosed with metastasis (14.8%). The more LNs were removed, the more positive LNs were detected. In Group 1 the average number of removed LNs was 18.0 (9–26), 15% of which were positive. In Groups 2 + 3 we removed an average of 16.6 (2–36) LNs in the salvage procedure, and 14.7% of these were positive. In contrast, the average number of removed LNs per primary RP was 13.3 (3–26), and 2.65% of these were positive (Figure 3).

**Figure 4:**
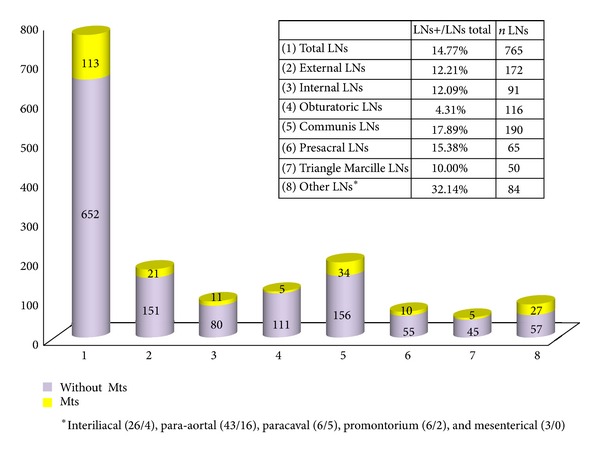
Localisation of LN metastasis (*n* = 41). Average of removed LNs per operation: 16.6. *n* = 765 (LNs in total).

The distribution of LN metastases from all SePLNDs regarding the location from which they were removed is shown in Figure 4. During salvage surgery, the largest percentage of (pelvic) LN metastases was found in the A. iliaca communis LNs and the presacralis area (total 17.9% and 15.4%, resp.) but closely followed by the region of the A. iliaca externa (12.2%), A. iliaca interna (12.1%), and the Marcille triangle (10.0%). The least frequent detection of metastases was in the obturator LNs (4.3%). Although a relatively large number of nodes (116) were removed from this area—which is standard in LND in PCa—the percentage of LN metastases was very low. More LNs were only removed in the A. iliaca communis area (190) and the A. iliaca externa (172), however with a high percentage of positive LNs (17.9% and 12.2%, resp., as mentioned above).

Para-aortal LN dissection was performed in 13 of 41 (31.7%) patients. We extended the area of LN dissection due to the positive PET-CT scan in the para-aortal area. In this particular group, metastases were found in 27 out of 84 removed LNs (32.1%).

**Figure 5:**
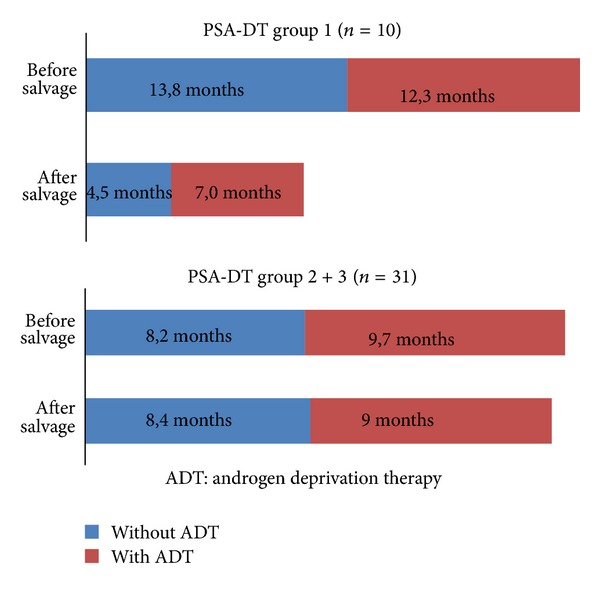
Dynamic of PSA-DT in months.

We considered the postsalvage PSA-DT and PSA value as the primary indexes of treatment efficiency regarding BCR-freedom. Moreover, we compared the PSA and PSA-DT before and after salvage surgery to document the effect of the SePLND on the clinical regression of the disease. Thus, a biochemical response (BR), which was defined as a PSA-value decrease after salvage surgery, was observed in 31 (75.6%) of 41 patients (Group 1: 70.0%, Group 2: 82.1%, and Group 3: 66.6%, resp.). We also attempted to evaluate the influence of ADT on the PSA-DT level. These data are shown in Figure 5 and Table 1. Indications for ADT were BCR or quick PSA-DT (individually but no longer than 6 months). In total, 30 (73.2%) patients had received ADT after primary treatment.

**Figure 6:**
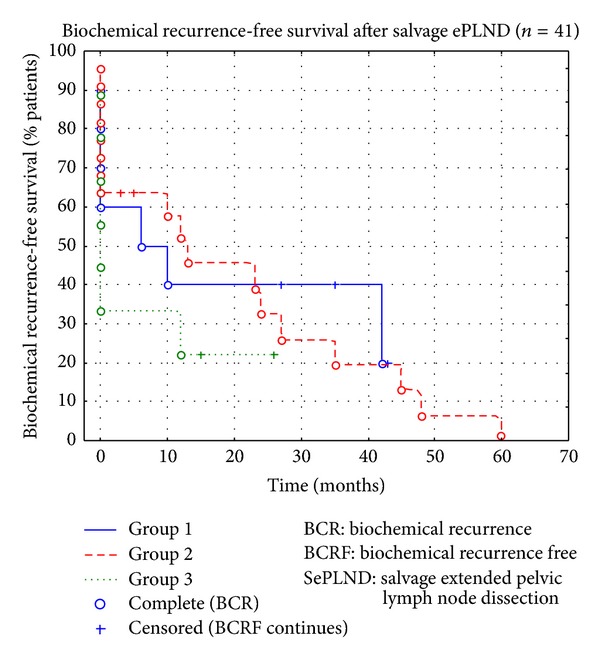
Kaplan-Meier curve with analysis of BCR-freedom survival in 3 groups (*n* = 41).

The BCR-free period in Group 1 was 6–43 months in 6 out of 10 (60%) patients (mean BCR-free period 27.2 months); in 3 patients of this group the BCR-free period (35, 27, and 43 months) has continued until the time of analysis (Figure 6 and Table 1).

During a follow-up period of two years or more, 14 of 22 (63.6%) patients from Group 2 remained without BCR (median BCR-free period in this group 17.5 months). In 5 patients the BCR-free period is still continuing (2; 3; 3; 5; 12 months). In the majority of these patients the follow-up and therefore their BCRF is only 2–5 months so far (Figure 6 and Table 1).

Three patients from Group 2 underwent a secondary sRT after SePLND. We decided to analyze these 3 cases further. It appears that all of them sooner or later started ADT treatment, and all of them had a BCR-free period of 35–45 months (mean 40.7 months). One of these patients unfortunately died of pancreas cancer, despite good PCa control.

In Group 3, 3 of 9 patients (33%) had a BCR-free period of 12–26 months (mean BCRF period in this group 17.7 months); 2 of these patients continue to have a BCR-free period until the moment of analysis (15 and 26 months, resp.). In all of them, the beginning of the BCR-free period coincides with ADT therapy (Figure 6 and Table 1).

The patients remained under our follow-up supervision after the salvage operation. According to the IIEF and IPSS scores, none of the patients reported a decrease of the quality of life after salvage ePLND. There was no relevant difference between the different patient groups regarding the IIEF and IPSS scores before and after operation.

In total, BCR-freedom was observed in 23 of 41 patients (56.1%) after salvage surgery. 75.6% of all patients showed a biochemical response, that is, a decrease of the PSA-level after SePLND. We analyzed all groups statistically and combined our data in a Kaplan-Meier curve (Figure 6). The mean BCR-free survival in all three groups (*n* = 41) was 21.4 months (95% CI 16.768–26.024; standard error 2.361).

Cancer-specific survival (CSS) and Kaplan-Meier analysis were performed for all three groups also. CSS analysis includes only those patients, whose follow-up was more than 24 months (*n* = 23). None of our patients died from PCa-specific causes during follow-up. Therefore, the absolute CSS is formally 100%, but of course there is no statistical reliability due to the limitations of this study. A small number of patients also cause the wide margins of the 95% confidence interval of 5-year CSS = 66.97%–100%; hazard ratio = 0.006683 (95% CI = 0–0.006683); median CSS = 103.7 months. It is statistically irrational to calculate CSS for each group because of the small number of patients included in the study.

We have observed relatively few complications associated with the salvage intervention/SePLND. We distinguished early (intraoperative/<3 days after surgery) and late (postoperative) complications. Early complications include 2 bleedings (causing relaparotomy) and 1 ureteral injury. Late complications include 1 rectovesical fistula, 2 lymphoceles, and 1 ureteral stricture (Table 2).

In addition, we analyzed the most severe complications. Intraoperative ureteral injury occurred in one patient, caused by difficulties with isolation of the ureter due to a suspected metastatic LN in the projection of the ureter. Prompt diagnosis of the iatrogenic injury led to immediate ureter neoimplantation by Boari technique and intraoperative double-J catheter-stent (6.32 Ch) placement. Thereby, we were able to avoid more serious complications, such as an otherwise necessary reoperation.

A rectovesical fistula was diagnosed in one patient with RT as a primary treatment after sRP/SePLND. One month after operation, this patient required a partial resection of the anterior rectum wall with insertion of a protective double sigmoidostomy. Abdomen-perineal closure of the fistula with suprapubic bladder drainage was performed one week later. Salvage surgery in patients who underwent primary RT can increase the risk of such complications and can lead to intraoperative difficulties due to the tissue-changing properties of radiation.

## 4. Discussion

The presented data support our hypothesis that ePLND in general and SePLND in particular can improve BCR-free survival. 21 of 41 patients (51.2%) were shown to have one or more positive lymph nodes during SePLND. The data from the last years shows how important it is to further extend PLND by additional dissection areas and to broaden the indication for salvage treatment [1, 2]. We believe that the frequently performed quick removal of fatty tissue from the fossa obturatoria is definitely not sufficient and should not go under the name of PLND let alone ePLND.

In one of our former studies we identified a correlation between the GS and the chances of finding positive LNs in sentinel PLND. In patients from high- and intermediate-risk groups the chance to detect one or more positive LNs was 30% [13]. Moreover, the number of positive LNs is an important factor for tumor-specific survival. Data from Bader and colleagues show a correlation between survival and the number of positive LNs. Of the 39 patients with only one positive node, 15 (39%) remained without signs of clinical or chemical progression, while only 6 out of 49 (12%) patients, who had at least two positive nodes, remained disease-free [14].

All in all, our presented data support the hypothesis that ePLND and SePLND, respectively, can improve BCR-free survival. This is confirmed by a study from Allaf and colleagues, who compared ePLND and limited PLND (lPLND). The 5-year PSA progression-free rate was 43% for ePLND versus 10% for the lPLND (*P* = 0.01)—despite the still relatively small number of removed LNs in the ePLND group (mean 11.6 versus 8.9, *P* < 0.0001) [15]. These results are also supported by an earlier study by Heidenreich et al. on 103 patients who underwent RP with extended PLND versus 100 patients after RP with standard lymphadenectomy. In the ePLND group, 28 lymph nodes (range 21 to 42) were dissected on average. Metastases were found in 27 of the 103 patients (26.2%) who underwent the extended procedure but only in 12 of the 100 patients (12%) who underwent the standard procedure (11 LNs dissected on average) [16].

Our own study on 106 intermediate and high-risk cancer patients who underwent ePLND clearly points to the necessity to remove at least 20 LNs or more to achieve an adequate oncologic outcome [1].

Why is 20 a threshold value? Is it the only one criterion for defining PLND as extended? Of course, 20 is only a relative number and the actual number of removed lymph nodes can also depend on other factors like the condition of the patient, the experience of the surgeon, or the priorities and specifics of different surgical schools [17]. But it is useful to fix a figure that can be used as an orientation mark regarding the quality of ePLND. However, it is not the only objective criterion. According to the definition proposed by us, ePLND includes dissection of LNs in the following areas: (1) in the fossa obturatoria, (2) along the external iliac artery, (3) parasacrally, (4) along the internal iliac artery, (5) along the common iliac artery, 3 cm above the bifurcation, (6) in the retroiliacal area, that is, the so-called “triangle Marseille,” and (7) in the preprostatic area [1]. Moreover, SePLND can be expected to have a positive oncological outcome for patients with BCR. This is confirmed by the data of a recent international retrospective, multi-institutional cohort analysis. There was a median follow-up of 4.4 yrs after sRP performed on 404 men with PCa recurrence post-RT from 1985 to 2009. According to this study, freedom from clinical metastasis was observed in >75% of patients 10 yrs after surgery. Patients with lower PSA levels pre-sRP and lower postradiation GS have the highest probability of a long-term cure from sRP [7].

A multimodality mapping study based on 34 patients (overall 317 LNs were detected) showed that PLND for PCa should include not only the external and obturator regions including the portions medial and lateral to the internal iliac vessels, but also the common iliac LNs at least up to the ureteral crossing, thus removing approximately 75% of all nodes that might potentially harbour metastasis [18].

We propose that SePLND should be performed according to the following principles: (1) LN dissection requires much care and small surgical steps. (2) The surgeon should not operate under time pressure. (3) The surgical access must always be transperitoneal. (4) Clearly distinguishable structures like the iliac vessels need to be identified safely before beginning with the dissection. (5) LN dissection should be performed systematically from top downwards. (6) The ureter must be identified safely and separated from the underlying pelvic vessels carefully and without injury, before starting the PLND. (7) Small or medium clips should be used to avoid extensive ligation as badly controlled movements during ligation can lead to damage of small lymph vessels. (8) Utilization of a tissue sealing system like the harmonic scalpel can safely seal LN vessels and shorten the operation time considerably.

These changes allowed us to achieve the following: (a) Group 1 (combined sRP + SePLND after RT) demonstrates an average BCR-free period of 27.2 months in all patients, and 20 out of 41 patients still have PSA levels of <0.5 ng/mL; (b) Group 2 (SePLND after RP) demonstrates BCR-free period of 17.5 months on average, and 29.4% of the patients still have PSA levels <0.5 ng/mL; (c) Group 3 (SePLND after RP and RT) demonstrates BCR-free period in 33.3% of the patients with an average duration of 27.2 months, and 2/3 of these still have PSA level <0.5 ng/mL. There were no patients with a rush increase of PSA level immediately after SePLND. Unfortunately, there are no reliable criteria of BCR after salvage treatment. These will have to be defined through the multicenter prospective randomized studies.

We found LN metastases in 15% of the removed LNs during sRP + SePLND and in 14.7% during SePLND procedures. Therefore, the probability of finding metastases during salvage surgery (summary sRP and SePLND) was nearly 5-6 times higher than in primary RP (2.65%) (Figure 3). Thus, during primary RP only 13.3 (3–26) LN were removed on average, only 2.65% of which were positive. These and our previous studies are strong arguments to perform ePLND even during primary RP, especially in intermediate and high-risk patients [12]. These data in conjunction with the BCRF periods show that most patients with LN metastases can live 2 years or more without BCR after salvage surgery of recurrent PCa; in other patients, the PSA progress is at least slowed down. As a result, the time until ADT initiation and thus the moment of castration resistance can be postponed. The quality of life is better in patients after salvage ePLND; omission of this procedure cannot be justified by fear of complications.

As mentioned above, 30 (73.2%) patients received ADT after primary treatment. In case of BCR after salvage surgery, ADT is often seen as the only treatment option. Consequently, sooner or later all the patients receive ADT to maintain or prolong the BCR-free condition after salvage surgery. We analyzed the sensibility to ADT after salvage surgery.

We observed an interesting dynamic of the PSA-DT in Group 1 (*n* = 10) before and after sRP + SePLND in relation to ADT presence/absence (Figure 5). *Prior to* sRP + SePLND, the PSA-DT was 13.8 months and thus higher *before* ADT was initiated compared to 12.3 *after* it was initiated. *After* sRP + SePLND, by contrast, the PSA-DT was lower *before* ADT was initiated (4.5 months) than after (7.0 months). Thus, *before* sRP + SePLND, the ratio of PSA-DT *with* versus *without* ADT was 0.89. *After* sRP + SePLND, by contrast, this ratio was 1.56. In conclusion, the ratio of PSA-DT with/without ADT after sRP + SePLND is 1.74 times higher than before sRP + SePLND. This supports the hypothesis that sensibility to ADT can be reestablished and the PSA-DT can be prolonged by means of salvage surgery; at the same time, the point at which the hormone-refractory condition occurs can be postponed. It must be conceded, however, that the correlation of PSA-DT with/without ADT after/before SePLND, respectively, was approximately equal in Groups 2 + 3.

Considering the whole patient cohort, 56.1% were able to achieve BCRF after SePLND with an average duration of 21.4 months. Moreover, it has been shown that it is possible for hormone-refractory patients to achieve BCRF-condition after SePLND, with or without ADT. As the duration of BCRF is limited, we must conclude that SePLND cannot be seen as a radical curative option, but it allows for prolonging the time until ADT has to be started or started again.

In general, the number of patients undergoing salvage treatment of PCa is very small, which is the main limitation of this study. Despite being a single center though, we have been able to involve a relatively large cohort of 41 patients over the last 7 years, which has encouraged us to standardize the surgical technique and persuaded us to expand the indication for salvage surgery.

When we planned our study, we distinguished 3 patient groups with different primary treatments in order to establish whether there would be any difference regarding the efficiency of salvage surgery with SePLND. The results regarding the biochemical response appeared to be similar in all three groups and all groups showed good results regarding BCR-free survival. We will continue to build up our databank and evaluate the long-term outcome.

There is no doubt that present data should be regarded as a preliminary experience of a limited series of selected patients. The limitation factors of this study are the number of patients, the single-center retrospective design and lack of a control group. These circumstances, however, apply to all prostate cancer centers due to the low incidence of salvage-surgical treatment for patients with recurrent PCa which has been confirmed by our analysis of the literature. Our data will be included in a large multicenter EAU study, the results of which will hopefully allow us in future to base the performance of SePLND in patients with recurrent PCa on valid multicenter data.

## 5. Conclusions

Salvage surgery is not just a “PSA cosmetic,” but an efficient alternative and a valid treatment option for patients with PCa recurrence. SePLND is a feasible and comparatively safe treatment option. In our cohort, it led to BCR-freedom in 56.1% of the patients with a mean duration of 21.4 months.

Furthermore, the ratio of PSA-DT with/without ADT after sRP + SePLND is nearly twice as high as the ratio before. Thus, the occurrence of a hormone-refractory condition in patients with BCR can be postponed; sensibility to ADT can be reestablished as well as prolonged by means of salvage surgery.

SePLND is an effective and relatively safe treatment option for selected patients with a manifest PCa recurrence. The main clinical outcome of SePLND is not the radical cure or remission of PCa but the achievement of a BCR-free condition as well as a restoration and prolongation of ADT sensibility and probably also a prolongation of CSS. This hypothesis requires further confirmation through analysis of CSS after appropriate follow-up. Multi center prospective studies should be performed to expand the base of evidence.

## Figures and Tables

**Table 1:** Results after salvage treatment in all three groups.

	*n*	Period of BCR-freedom %	BR %	Mean BCR-free period (months)	Patients with postsalvage ADT
Group 1	10	60.0%	70.0%	27.2	40.0%
Group 2	22	63.6%	82.1%	17.5	40.7%
Group 3	9	33.3%	66.6%	17.7	44.4%

Total	41	56.1%	75.6%	21.4	41.5%

**Table 2:** Complications in salvage treatment (*n* = 41).

Complication	Frequency
Early (intraoperative or 3 days after surgery)	
Bleeding	2 (4.9%)
Ureteral injury	1 (2.4%)

Late (more than 3 days after surgery)	
Lymphocele	2 (4.9%)
Ureteral stricture	1 (2.4%)
Rectovesical fistula	1 (2.4%)

## Take Home Messages

1. Extended salvage PLND in patients with LN metastasis and BCR of PCa is a valid and safe therapy option. We experience long periods of biochemical control in more than 50% of the patients and relatively small numbers of salvage surgery complications.
2. Extended salvage PLND is not a curative treatment option but it increases the duration of ADT sensibility as well as the BCRF and CSS.
3. The ratio of PSA-DT with/without ADT after sRP + SePLND is almost twice as high as before sRP + SePLND.
4. The technique of primary and salvage extended PLND should be standardized and accepted as a necessity in intermediate- and high-risk PCa patients as well as in patients with PCa relapse.
